# Geospatial patterns in terrestrial organic matter reactivity across four shelf seas spanning the Eurasian Arctic

**DOI:** 10.1126/sciadv.adt6806

**Published:** 2025-09-10

**Authors:** Junjie Wu, Felipe Matsubara, Gesine Mollenhauer, Ruediger Stein, Bingbing Wei, Kirsten Fahl, Xiaotong Xiao, Örjan Gustafsson

**Affiliations:** ^1^ Department of Environmental Science, Stockholm University, Stockholm, Sweden.; ^2^ Bolin Centre for Climate Research, Stockholm University, Stockholm, Sweden.; ^3^ Alfred Wegener Institut Helmholtz Zentrum für Polar und Meeresforschung, Bremerhaven, Germany.; ^4^ MARUM Center for Marine Environmental Sciences and Faculty of Geosciences, University of Bremen, Bremen, Germany.; ^5^ Frontiers Science Center for Deep Ocean Multispheres and Earth System, and Key Laboratory of Marine Chemistry Theory and Technology, Ocean University of China, Qingdao, China.

## Abstract

Organic matter stored in Arctic permafrost represents a key component of the carbon cycle, yet its reactivity across heterogeneous continent-scale permafrost regions remains poorly understood. Here, we leverage the four shelf seas of the Eurasian Arctic as integrative receptor systems to evaluate terrestrial organic matter reactivity, assessed by examining organic carbon preservation as a function of ^14^C-constrained cross-shelf transport time. Our findings reveal higher reactivity of terrestrial organic matter released to the Laptev Sea and the eastern East Siberian Sea, lower reactivity in the western East Siberian Sea, and no deducible degradation in the Kara Sea. The reactivity of terrestrial organic matter is primarily determined by the degradation status and composition of its source, alongside potential microbiological controls during transport. This study reveals the heterogeneity of terrestrial organic matter reactivity across the Eurasian Arctic margin and highlights the need for detailed assessments of region-specific carbon release and modeling parameterization.

## Teaser

The reactivity of terrestrial organic matter varies across the Eurasian Arctic shelf seas, affecting carbon cycle dynamics.

## INTRODUCTION

Permafrost holds ~50% of the global soil organic carbon (OC) (*1*), constituting a reservoir that exceeds the atmospheric carbon pool by about 60% (*2*). Periods of permafrost formation effectively inhibit microbial decomposition of organic matter (OM) and therefore lead to a buildup of soil OM, which may subsequently trigger increased greenhouse gas release upon permafrost thaw (*3*–*5*). Emerging studies in marine receptor systems, often using source-specific molecular biomarkers, provide insights on the behavior and fate of permafrost-exported OM integrated over large spatial scales (*6*–*13*). However, most molecular biomarker–based studies have been geographically limited to parts of individual shelf seas, such as either the Laptev Sea, East Siberian Sea, or Beaufort Sea. The restricted molecular fingerprinting of terrestrial OM at a continental scale, coupled with a lack of constraints on its transport time, has hindered our ability to quantitatively determine the reactivity of OM exported from permafrost and to identify the factors influencing carbon fate during cross-shelf transport.

The reactivity of OM, commonly defined as its susceptibility to decomposition, is often inferred from its radiocarbon age (*14*), although this relationship becomes more complex in permafrost-affected environments (*12*). In general, older ^14^C ages indicate that OM has experienced longer residence time in the environment without substantial decomposition, reflecting its stability and lower reactivity. In contrast, younger ^14^C signatures reflect more recently fixed carbon with limited exposure to transformation or storage and are thus usually considered more reactive. However, in permafrost systems, for OM that stays frozen for extended periods with a higher initial reservoir age, an old carbon age does not necessarily indicate low OM reactivity. The Circum-Arctic Sediment Carbon Database (CASCADE), covering more than 4000 locations across the Arctic shelf seas, shows lower Δ^14^C values in the eastern Eurasian Arctic Ocean (sourced from continuous permafrost zones) than in the western Eurasian Arctic Ocean (sourced from more discontinuous permafrost zones) (*7*). Similarly, continental-scale studies of the Eurasian Arctic river mouths demonstrate that OM exported to the eastern Eurasian Arctic Ocean is older but less degraded, while OM exported to the western Eurasian Arctic Ocean is younger but more degraded (*12*, *15*, *16*). These findings suggest that the permafrost systems introduce a complexity between carbon age and reactivity, highlighting the need for more direct assessments of the reactivity of permafrost-exported OM beyond radiocarbon age alone.

The reactivity of OM can be shaped by a combination of selective microbial degradation, organic-mineral interactions, and structural characteristics of the molecules. Microorganisms tend to preferentially degrade labile components of carbon pools, leaving behind recalcitrant components (*17*), which substantially alters OM reactivity. Another critical process for OM stabilization is the sorptive removal, where key studies have highlighted the importance of mineral protection of natural OM (*18*–*22*). The interactions between OM and the mineral surface are facilitated by the adsorption sites available on the mineral surface (*19*–*21*). Iron minerals such as iron oxides and iron oxyhydroxides, in addition to clay minerals, also contribute to OM stabilization because of their high sorption capacity (*22*–*24*). Furthermore, the chemical structure of OM itself influences reactivity. Black carbon, a highly recalcitrant form of OC from incomplete combustion of biomass and fossil fuels, is an important terrestrial component in the East Siberian Arctic Shelf, with about 101 kilometric tons year^−1^ exported by Eurasian rivers (*25*) and contributing relatively uniform concentrations across the East Siberian Arctic Shelf sediments (*26*). Petrogenic carbon, another refractory form of OM, originates mainly from the Canadian Arctic and off Svalbard, whereas only limited inputs have been observed in the East Siberian Arctic Shelf seas and Kara Sea (*7*, *9*, *12*). Together, the reactivity of OM is governed by complex and interacting factors. Understanding the geographic differences and mechanisms that control OM reactivity is essential for better assessing its potential impact on the carbon cycle.

Estimation of cross-shelf transport time is challenging yet enables a quantitative elucidation of the reactivity of permafrost-exported OM and provides the foundation for exploring the underlying controlling mechanisms. Large-scale quantitative assessments of reactivity of permafrost-exported OM were pioneered in the Laptev Sea (*6*). By using compound-specific radiocarbon analyses of terrestrial biomarkers to determine cross-shelf transport time, Bröder *et al.* (*6*) successfully constrained a system-average ambient degradation rate constant [2.8 ± 0.2 per thousand years (kyr^−1^)] for terrestrial OC across the Laptev Sea. Building on this approach, Bröder *et al.* (*27*) extended the relationship between transport time and water depth from the Laptev Sea to the East Siberian Sea, estimating a degradation rate constant of 1.5 kyr^−1^ for the broader region encompassing both the Laptev Sea and East Siberian Sea. These studies established an approach for quantifying terrestrial OC degradation and provided first insights into large-scale OM reactivity through ambient measurements. However, these studies were conducted on a limited spatial scale and were unable to conclude about the controlling mechanisms. Given that cross-shelf transport time has been quantified for the Laptev Sea (*6*), is less constrained for the East Siberian Sea (*27*), and remains unconstrained for the western Eurasian Arctic Shelf Seas (e.g., Kara Sea), dedicated regional determinations of cross-shelf transport time are warranted for broader continent-scale investigations and a refined understanding of this complex carbon dynamics system.

In this study, we aim to assess the reactivity of permafrost/terrestrially exported OM across four shelf seas in the Eurasian Arctic and to explore primary controlling mechanisms. To this end, we first established a regional relationship between terrestrial carbon age and age-derived cross-shelf transport time for the Kara Sea. By analyzing total OC (TOC), carbon isotopes (δ^13^C and Δ^14^C), mineral specific surface area (SSA), lipid biomarkers, and lignin phenols in surface sediments along a Kara Sea transect, we were able to investigate OM sources, decomposition status, and decomposition processes. In addition, we refined the estimates of transport time for the East Siberian Sea, addressing limitations associated with the earlier approach. Last, we compared the reactivity of permafrost-exported OM in the Kara Sea, Laptev Sea, river-influenced western East Siberian Sea (WESS), and Pacific-water-influenced eastern East Siberian Sea (EESS), providing a continental-scale perspective on the reactivity of exported terrestrial OM and its controlling mechanisms.

## RESULTS

### Source apportionment of OC in the Eurasian Arctic shelf

**Fig. 1.**
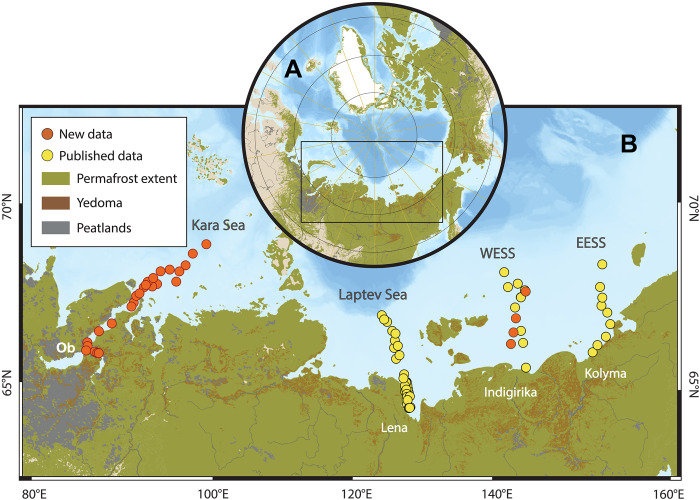
Overview of the Arctic and sampling sites in the four Eurasian Arctic shelf seas. (**A**) Overview of the Arctic and the study area (black box); (**B**) sampling sites in the Kara Sea and WESS (new data, red dots) and earlier published data (yellow dots), both sets combined and interpreted within an integrated framework in the current study. The Arctic Ocean base map is based on IBCAOv4 (*61*).

**Fig. 2.**
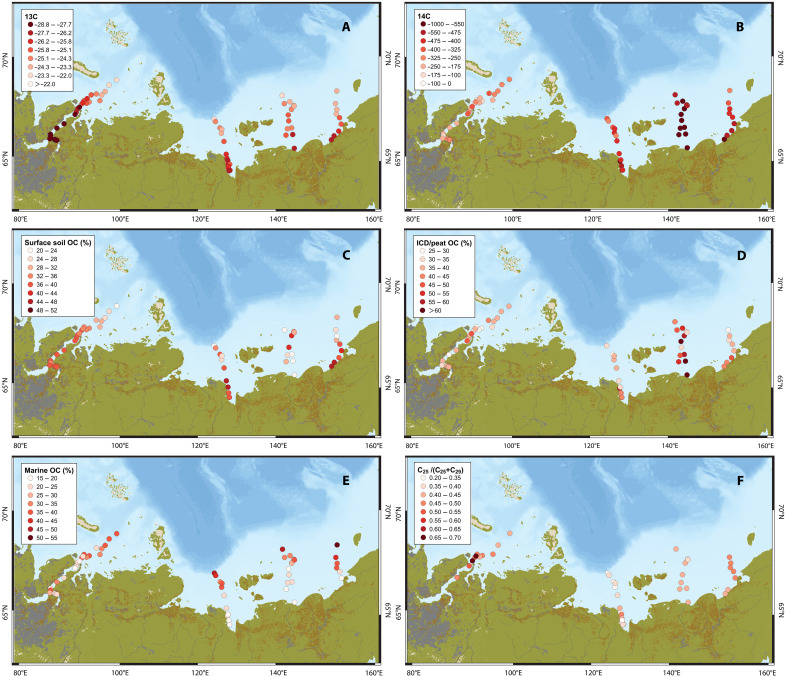
Organic-geochemical information on the sources of terrestrial OM across the four Eurasian Arctic shelf seas. (**A**) δ^13^C value of bulk OC; (**B**) Δ^14^C value of bulk OC; (**C**) surface soil/AL OC fraction; (**D**) ICD/peat OC fraction; (**E**) marine OC fraction; (**F**) sphagnum indicator proxy *n*-alkane C_25_/(C_25_ + C_29_).

Stable carbon isotope (δ^13^C) and radiocarbon (Δ^14^C) signals of bulk OC provide an overview of carbon sources for the Eurasian Arctic shelf seas (Fig. 1). The OC-δ^13^C reveals a spatial pattern in OC composition across all transects, with a dominance of terrestrial OC (lower δ^13^C values) along coasts and a relative increase in marine OC (increasing δ^13^C values) seaward (Fig. 2). The Δ^14^C demonstrate relatively higher values along the Kara Sea transect and relatively lower values along the WESS transect (Fig. 2). This can be explained by the terrestrial OM in the Kara Sea originating from a discontinuous permafrost region, where the mixture of younger OM from nonpermafrost soils and older OM from permafrost soils leads to a shorter apparent mean residence time and consequently less ^14^C depletion (*12*). In contrast, the dominant source of terrestrial OM in the WESS is associated with coastal erosion of the ice complex deposit (ICD) which was formed during the Late Pleistocene and thus exhibits greater ^14^C depletion (elaborated below) (*7*, *9*, *11*, *28*).

Source apportionment combining δ^13^C and Δ^14^C serves as a powerful tool to quantitatively constrain OC sources in the Eurasian Arctic shelf seas. This is here achieved for the four transects, spanning across the entire Eurasian Arctic margin, with three possible OC source classes, i.e., terrestrial from the active layer (AL), ICD, and autotrophic marine OC. Given that Kara Sea receives substantial material derived from peatland (*29*), indicated by the sphagnum indicator [C_25_/(C_25_ + C_29_)] (Fig. 2F), we employed surface soil and subsurface peat, instead of AL and ICD, as end-members specifically for the Kara Sea transect (see Materials and Methods) (*7*). Notably, while autochthonous particulate OM (POM) from rivers is generally considered a minor source of OM to Arctic shelf sediments (*7*, *16*, *28*), studies by Behnke *et al.* (*30*) and Ogneva *et al.* (*31*) proposed that aquatic biomass may represent a substantial fraction of Arctic riverine POM. We acknowledge that riverine plankton–derived OC was not explicitly included as a separate end-member in our model, which may introduce some uncertainty to our source apportionment. The outcome is broadly consistent with the interpretation from just stable carbon isotopes, affirming more terrestrial OC closer to shore and increased marine OC contribution seaward (Fig. 2). In particular, although the WESS is a receptor of terrestrial OM from the Indigirka River, source apportionment suggests that this system serves primarily as a hotspot receptor for ICD-OC from coastal erosion, while the riverine input of the Indigirka River (AL-OC) is smaller (Fig. 2), aligning with previous findings (*9*, *27*, *28*).

Overall, the carbon isotope-based source apportionment reveals a substantial contribution of terrestrial OC nearshore, with a marked shift toward marine OC further seaward. Notably, the WESS emerges as a key receptor for ancient ICD-OC from coastal erosion rather than carbon from river discharge.

### Degradation status of terrestrial OM across the Eurasian Arctic shelf

**Fig. 3.**
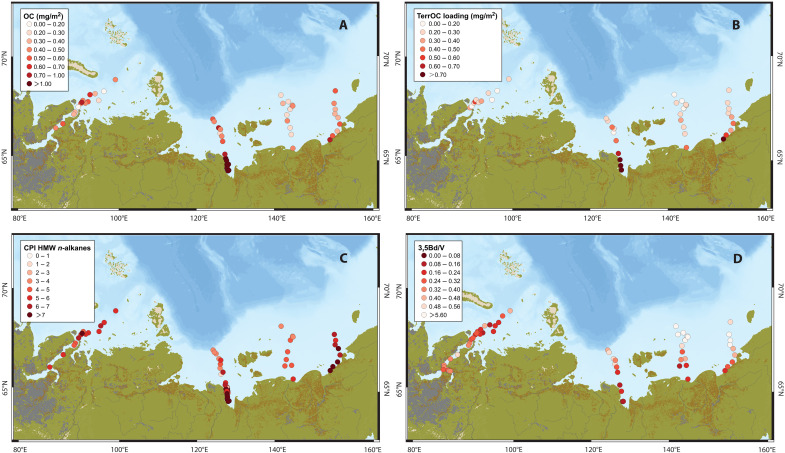
Geochemical information for assessing the degradation status of terrestrial OM across the four Eurasian Arctic shelf seas. (**A**) OC content; (**B**) SSA; (**C**) lipid-based degradation proxy—the CPI of high-molecular-weight (HMW) *n*-alkanes; (**D**) lignin-based degradation proxy 3,5Bd/V.

The terrestrial OM decomposition status varies markedly across and between the different Eurasian Arctic shelf seas. The degradation proxy carbon preference index (CPI) of high-molecular-weight (HMW) *n*-alkanes exhibits decreasing patterns (more degraded) seaward in the Laptev Sea and EESS. In contrast, this degradation pattern is absent in the Kara Sea and WESS (Fig. 3), similar to previous findings from the Kara Sea (*32*). A similar lack of trend is observed for the 3,5Bd/V (3,5-dihydroxybenzoic acid/vanillylphenol ratios) proxy in the Kara Sea, while degradation patterns with increasing values (more degraded) seaward are observed in the Laptev Sea, WESS, and EESS (Fig. 3). Notably, the Kara Sea stands out with no discernable degradation across all these molecular degradation proxies.

**Fig. 4.**
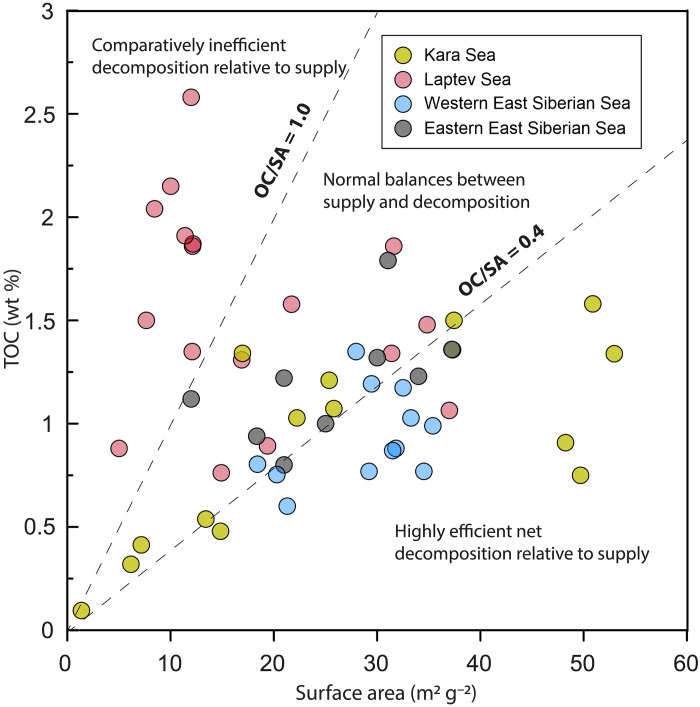
TOC content as a function of mineral SSA in the surface sediment of Eurasian Arctic shelf seas. The SSA-normalized OC loading ratios between 0.1 and 0.4 mg C m^−2^ usually reflect highly efficient net decomposition relative to supply. Values between 0.4 and 1.0 mg C m^−2^ suggest normal balances between supply and decomposition. Values >1.0 mg C m^−2^ indicate comparatively inefficient decomposition relative to supply.

The global observation suggests that the SSA-normalized OC loading ratios usually reflect balances between OC supply and decomposition. Low ratios (0.1 to 0.4 mg C m^−2^) often indicate regions of highly efficient net decomposition relative to supply, while intermediate ratios (0.4 to 1.0 mg C m^−2^) suggest normal balances between supply and decomposition in rivers, estuarine, and shelf environments. In contrast, high ratios (>1.0 mg C m^−2^) may indicate regions of comparatively inefficient decomposition relative to supply (*33*). In this context, the SSA-normalized OC loadings observed at the river or estuary mouths suggest intermediate OM decomposition upon delivery to the Kara Sea and WESS (0.5 mg C m^−2^) and inefficient OM decomposition upon delivery to the Laptev Sea and EESS (2.8 mg C m^−2^ to the Laptev Sea and 2.5 mg C m^−2^ to the EESS) (Figs. 3 and 4).

### Quantitative constraints on transport times

Determination of transport time opens the possibility to quantitatively constrain intersystem differences in large-scale ambient OM degradation. The general approach to estimate transport time was pioneered by Bröder *et al.* (*6*) using the ^14^C ages of specific terrestrial biomarkers to put time constraints on the net cross-shelf transport of terrestrial OM. In their study, the ^14^C ages systematically increased across the Laptev Sea shelf. The authors related the ^14^C ages to both the water depth and offshore distance. This approach aims to reduce the influence of measurement uncertainty and/or complex matrix that can otherwise result in apparent reversals in the offshore ^14^C age trend. The regression models show good linear correlations in both cases. The authors chose to focus on the relationship between ^14^C age and water depth to derive transport time on their main discussion. On the basis of this concept, we note that both depth- and distance-based regressions are valid approaches, and the selection between them should be guided by the regional characteristics and shelf morphology. In our study, slightly different but conceptually consistent methods were applied across the various shelf seas. For each transect, we selected the innermost station as the reference starting point and calculated the relative water depth and offshore distance for all other sites accordingly.

**Fig. 5.**
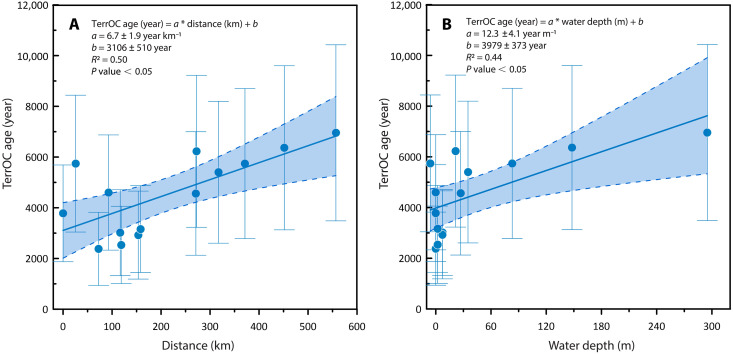
Relationships between terrestrial OC age and cross-shelf distance and water depth for the Kara Sea. With the innermost sites selected as reference, (**A**) ^14^C-derived terrestrial OC (TerrOC) age versus apparent distance and (**B**) ^14^C-derived terrestrial OC age versus apparent water depth. Error bars represent propagated uncertainties.

In the Kara Sea transect, bulk OC-Δ^14^C represents a composite signal of both terrestrial and marine OC sources. On the basis of δ^13^C signal and mass balance constraints, our approach isolated the relative contribution of the marine source to bulk OC. The Δ^14^C end-member values are reasonably well constrained for the marine OC, i.e., a Δ^14^C end-member value of −3 ± 55‰ (*n* = 11) for the western regimes of 160°E and a Δ^14^C end-member value of −50 ± 12‰ (*n* = 5) for the eastern regimes of 160°E because of a frontal zone with river-influenced waters and stronger ^13^C-depleted phytoplankton in the East Siberian Arctic Shelf (*9*). We correct for this marine Δ^14^C-OC contribution and thus deduced the terrestrial Δ^14^C-OC signal. The terrestrial Δ^14^C-OC signal was subsequently converted to ages of the terrestrial OC along the transect to provide a long-term-averaged cross-shelf transport time axis (Fig. 5).

The terrestrial OC age in the Kara Sea increases with both offshore distance and water depth, providing linear relationships for transport time estimates. Note that the water depth from inner Ob Bay to the mouth is not strictly increasing. This would introduce complexity to the relationship between terrestrial OC age and water depth. To address this, the relationship between terrestrial ^14^C-OC age and offshore distance and that between terrestrial ^14^C-OC age and water depth were established by excluding samples within the Ob Bay. Consequently, a linear relationship emerges between terrestrial OC age and offshore distance: Age (year) = *a* (year km^−1^) × Distance (km) + *b* (year) (*R*^2^ = 0.50, *P* < 0.05), with fitted parameters *a* = 6.7 ± 1.9 year km^−1^ and *b* = 3106 ± 510 years (Fig. 5A). Similarly, a linear relationship is observed for terrestrial OC age and water depth: Age (year) = *a* (year m^−1^) × Water depth (m) + *b* (year) (*R*^2^ = 0.44, *P* < 0.05), with fitted parameters *a* = 12.3 ± 4.1 year m^−1^ and *b* = 3979 ± 373 years (Fig. 5B). Using the innermost station (near the Ob Bay mouth) as a starting point, the Kara Sea transect yields a net transport time of 3730 ± 1060 years based on the offshore distance relationship or 3630 ± 1210 years based on the water depth relationship. These estimates are close to the cross-shelf transport time of 3600 years constrained by a similar approach for the similarly very wide Laptev Sea (*6*), suggesting a reasonable regional estimate for the Kara Sea.

Notably, a decrease in ^14^C ages was observed at shallow water depths (Fig. 5B), while the underlying cause remains unclear. Both preferential degradation and preferential deposition could potentially alter the composition of the terrestrial OM pools and consequently the ^14^C ages. However, the established mechanisms of terrestrial OM transport and degradation typically result in an increase in ^14^C age (*8*), contrary to the trend we observed, which makes this pattern difficult to explain. In addition, compared to the scattered data points in the water depth relationship, the distance-based relationship provides a more uniform distribution of data points along the *x* axis (Fig. 5, A and B), which enhances the stability and interpretability of the regression model. We therefore used the relationship between terrestrial ^14^C-OC age and offshore distance to estimate transport time for the Kara Sea.

These approaches to estimate transport time for the Laptev Sea and Kara Sea encounter limitations when applied to the East Siberian Sea. The compound-specific radiocarbon analysis of terrestrial lipid biomarkers that was used for the Laptev Sea (*6*) is not available for the East Siberian Sea. The only available radiocarbon age in the East Siberian Sea derives from bulk terrestrial Δ^14^C-OC. Despite the fact that we successfully used bulk terrestrial Δ^14^C-OC to estimate Kara Sea cross-shelf transport time, it is noteworthy that this application was under a circumstance of limited degradation. Degradation, particularly preferential degradation, may alter the average age of the remaining OC, complicating age estimates. Such processes cannot be ignored for the East Siberian Sea regions, rendering this approach inapplicable to the East Siberian Sea. In this context, an alternative is to adopt the established linear relationship from nearby regions.

A previous study has used the linear relationship between terrestrial OC age and water depth derived for the Laptev Sea also to East Siberian Sea transects (*27*). This yields transectual net transport times of 2520 years in the WESS and 1765 years in the EESS, which are somewhat less than that for the Laptev Sea. The apparently more rapid lateral transport could also result from less efficient degradation of a younger subfraction of terrestrial OM than in the Laptev Sea. Given the broad and shallow nature of the shelves across most of the East Siberian Sea, i.e., minimal changes in water depth over long distances, the relationship between terrestrial OC age and water depth appears less appropriate than a distance-based approach. The comparably good fit of terrestrial OC age versus offshore distance in the Laptev Sea seems a viable option for the East Siberian Sea. The distance-based transectual net transport time in the East Siberian Sea is 3000 to 3500 years, comparable to the Laptev Sea and Kara Sea. In this context, using the distance-based approach from the Laptev Sea is deemed more reasonable than the water depth-based approach. However, we acknowledge that transport times in the ESS are less well constrained because of limited region-specific data, and applying the Laptev Sea relationship introduces additional uncertainty.

Together, the terrestrial OM transport time for the Kara Sea and Laptev Sea is constrained based on their regional linear relationship between water depth and terrestrial OC ages and found to be similar. For the WESS and EESS systems, the estimates of transport times are derived from the Laptev Sea estimate using a distance-based regressional approach.

### Quantitative estimates of terrestrial OM reactivity and fate

Quantification of the large-scale ambient-system degradation rate of terrestrial OC in the recipient shelf seas helps in constraining ambient system-averaged OM reactivity. Here, we estimated terrestrial OC loadings, which were determined by multiplying bulk OC loadings by the terrestrial OC fractions (Fig. 3B). The terrestrial OC fractions were combined from the AL and ICD fractions and, for the Kara Sea, from the surface soil and subsurface peat fractions. The terrestrial OC loadings show exponential declines with increasing transport time. To characterize terrestrial OM reactivity during this dynamic process, we derived a first-order apparent system-wide degradation rate constant for terrestrial OC by fitting a first-order kinetics reaction function to these data

$$C\left(t\right)=C_{\text{deg}}\left(0\right)\times e^{-\mathrm{kt}}+C_{R}$$ (1)

where *C*(*t*) is the terrestrial OC loading at locations corresponding to a derived transport time *t*, *C*_deg_(0) is the initial loading of degradable terrestrial OC, *k* is the apparent first-order degradation rate constant, and *C*_R_ refers to the loading of recalcitrant OC (practically undecomposable OC), corresponding to the asymptotic value for *C* (*t* → ∞). The sum of degradable carbon *C*_deg_(0) and recalcitrant carbon *C*_R_ derived from the fitted equations, in principle, equals the initial bulk terrestrial OC *C*(0), i.e., *C*_deg_(0) + *C*_R_ = *C*(0). The degradation rate constant *k* quantifies the rate at which the degradable terrestrial OC degrades over time, thus describing terrestrial OM reactivity for any given system (shelf sea).

The parameter *C*_R_ also reflects terrestrial OM reactivity, albeit in a different manner than the degradation rate constant *k*. While *k* reflects how quickly the OC is being consumed or transformed, *C*_R_ represents a portion of the exported OC that is recalcitrant and remains in the system over long timescales, including components that have been identified in the East Siberian Arctic shelf system such as through physical protection (occlusion and sorption with FeOOH) (*22*) or low chemical reactivity (e.g., black carbon or kerogen) (*25*, *26*, *34*).

To facilitate the comparison of remobilized terrestrial OM reactivity in different sectors of the Eurasian Arctic margin, through a perspective of the recalcitrant terrestrial OC, we here introduce the concept of cross-shelf carbon preservation index (PI). This index metric is defined as the terrestrial OC loading at time *t* relative to the initial terrestrial OC loading observed at the innermost stations [*C*(0)′] of each individual transect, i.e., *C*(*t*)/*C*(0)′. The carbon preservation index thus follows this function

$$\text{PI}\left(t\right)=f_{\text{deg}}\left(0\right)\times e^{-\mathrm{kt}}+f_{R}$$ (2)

where PI(*t*) is the carbon preservation index at time *t*, *f*_deg_(0) is the fraction of degradable terrestrial OC, *f*_R_ is the fraction of recalcitrant terrestrial OC, and *k* is the removal rate constant. By using this function, the carbon preservation index at *t* = 0 [PI(0)] is normalized to 1, and the fractions of recalcitrant terrestrial OC *f*_R_ become comparable among the four transects. Given that the selection of *t* = 0 (innermost site) may potentially introduce artificial variability in the fitted residual fraction *f*_R_, we compared *f*_R_ values obtained from the normalized equation (Eq. 2) with those derived by dividing the absolute *C*_R_ from the original decay model by *C*(0) (Eq. 1). The results show that the *f*_R_ values are largely consistent across the two approaches, with only a minor deviation (7%) observed for the Kara Sea. This discrepancy does not affect the overall trends or interpretations in this study. It is also noteworthy that the carbon removal or degradation rate constant, denoted as *k*, maintains consistent values in both expressions. The resulting quantitative constraints on *k*, *f*_deg_(0), and *f*_R_ for the four different shelf sea systems are summarized in Table 1.

**Table 1.** Compilation of derived parameters for the cross-shelf reactivity of terrestrial OC. *P* values: Kara Sea (*P* > 0.05); all others (*P* < 0.05).

Eurasian Arctic shelf seas	*R* ^2^	*k* (kyr^−1^)	*C*(0) (mg m^−2^)	*C*_deg_(0) (mg m^−2^)	*C*_R_ (mg m^−2^)	*f*_deg_(0)	*f*_R_
Kara Sea	0.13	2.3 ± 4.4	0.30 ± 0.11	0.12 ± 0.08	0.18 ± 0.07	0.37 ± 0.25	0.56 ± 0.23
Laptev Sea	0.98	2.0 ± 0.4	1.73 ± 0.10	1.46 ± 0.08	0.27 ± 0.06	0.82 ± 0.05	0.16 ± 0.03
Western East Siberian Sea	0.58	1.2 ± 0.9	0.41 ± 0.07	0.20 ± 0.06	0.21 ± 0.03	0.47 ± 0.14	0.51 ± 0.07
Eastern East Siberian Sea	0.93	2.5 ± 0.7	0.83 ± 0.07	0.57 ± 0.06	0.26 ± 0.03	0.68 ± 0.07	0.31 ± 0.03

**Fig. 6.**
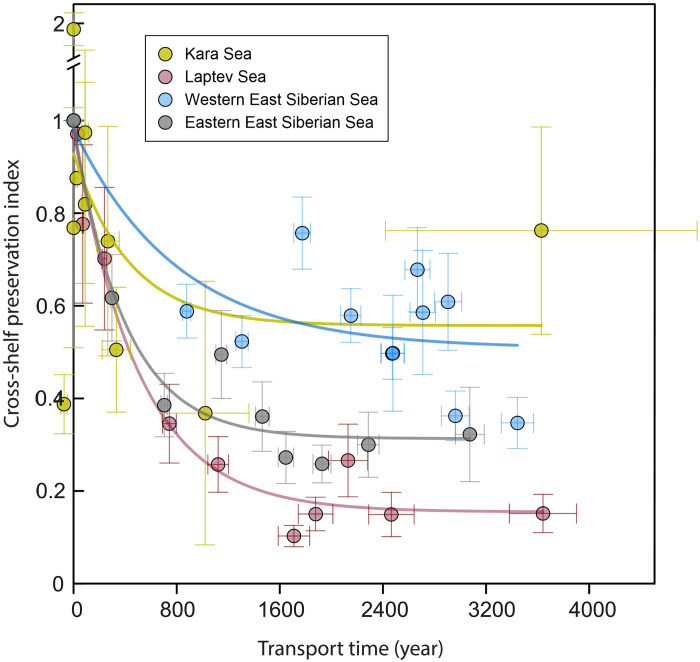
Terrestrial OM reactivity across transects in four Eurasian Arctic shelf seas. An exponential decay curve PI(*t*) = *f*_deg_(0) × *e*^−*kt*^ + *f*_R_ is fitted to the cross-shelf preservation index as a function of ^14^C-estimated transport time. The cross-shelf preservation index is defined as the terrestrial OC loading at time *t* relative to the initial terrestrial OC loading observed at the innermost sites of each transect [*C*(*t*)/*C*(0)′]. The asymptotic value for PI (*t* → ∞) = *f*_R_ is the recalcitrant fraction. Other fitted parameters describing the terrestrial OC reactivity can be found in Table 1. The transport time in the Laptev Sea and Kara Sea is calculated from the age versus water depth relationship, while in the East Siberian Seas, it is calculated from the age versus distance relationship. Horizontal and vertical error bars represent propagated uncertainties in transport time and cross-shelf preservation index, respectively.

These fitted parameters demonstrate differences in the reactivity of exported terrestrial OM between the different sectors of the Eurasian Arctic margin. The highest degradation rate constant of 2.5 ± 0.7 kyr^−1^ was found for the EESS, a moderate value of 2.0 ± 0.4 kyr^−1^ for the Laptev Sea, and the lowest value of 1.2 ± 0.9 kyr^−1^ for the WESS (Fig 6 and Table 1). The recalcitrant fractions of terrestrial OC are determined to be the largest (51 ± 7%) in the WESS, moderate (31 ± 3%) in the EESS, and smallest (16 ± 3%) in the Laptev Sea (Fig. 6 and Table 1). The most distinct result is that there is no discernible degradation pattern resolved for the Kara Sea (*R*^2^ = 0.13) (Fig. 6 and Table 1); the Kara Sea degradation rate constant and the recalcitrant fractions of terrestrial OC lack statistical significance (*P* > 0.05). This intriguing result based on the cross-shelf patterns of OC across the Kara Sea, in contrast to the results for the three other shelf seas, is consistent with the findings from the biomarker-based degradation proxies presented above. Accordingly, it can be inferred that the terrestrial OM exported across the Kara Sea remains largely unaltered during transit.

Together, this evaluation reveals relatively high terrestrial OM reactivity along the Laptev Sea and EESS transects, with lower terrestrial OM reactivity and limited degradation along the WESS transect and no detectable degradation of the exported terrestrial OM across the Kara Sea.

## DISCUSSION

Differences in terrestrial OM reactivity between the four large shelf seas spanning the northern seaboard of the Eurasian Arctic are likely determined by the degradation status of terrestrially exported OM. In general, the Laptev Sea and EESS are recipients of terrestrial material with higher SSA-OC loadings. In contrast, the Kara Sea and WESS receive permafrost/peat OM with comparatively lower SSA-OC loadings (Fig. 3). Although SSA-normalized OC loading operates independently from the degradation rate constant *k* and the recalcitrant fraction *f*_R_ in the equations above, it offers insights into the OM decomposition status, i.e., less degraded OM delivered to the Laptev Sea and EESS while more degraded OM to the Kara Sea and WESS (Fig. 4), which in turn influences these parameters.

These observations are corroborated by the molecular degradation proxies for terrestrial OM, CPI and 3,5Bd/V. These proxies consistently indicate a supply of less degraded OM to the Laptev Sea and EESS and more degraded OM to the Kara Sea and WESS (Fig. 3, C and D). Decomposition status may serve as a corollary for preferential utilization of multiple substrate pools within the total pool, thereby reflecting intrinsic reactivity-degradation characteristics of different carbon pools. It is deduced that less degraded OM delivered to the Laptev Sea and EESS thus likely includes more reactive substrates susceptible to remineralization, while the more degraded OM supplied to the Kara Sea and WESS likely contains more recalcitrant and less degradable substrates. In addition, on the basis of the dissolved OC (DOC) and particulate OC (POC) fluxes from pan-Arctic rivers (*35*, *36*), the DOC/POC ratio varies across the Ob River (7.2), Yenisey River (18.7), Lena River (7.0), and Kolyma River (6.7), indicating a higher proportion of DOC exported to the Kara Sea compared to the Laptev Sea and EESS. Previous studies have shown that POC is often more prone to degradation than DOC in the Arctic environments (*37*–*39*), possibly due to higher enzymatic rate, enhanced bacterial activities, and the greater ease with which microbes can colonize particulate substrates (*40*). Therefore, these variations in DOC/POC ratio may further support our observations regarding OM reactivity.

Permafrost type, thawing processes, and history may affect microbial physiological inhibition, which in turn could potentially influence the OM decomposition status. In the continuous permafrost zones, the persistently freezing conditions in the Lena River and Kolyma River catchments likely limit microbially mediated decomposition of OM that is exported to the Laptev Sea and EESS (*9*, *12*). In contrast, the Ob River catchment is situated in a discontinuous permafrost zone characterized by extensive peatland. Despite the peatland being recognized as a reservoir for OM accumulation because of the slow decomposition rates under waterlogged anoxic conditions (*41*), the more decomposed OM observed in the Kara Sea (*9*, *12*), however, implies pronounced microbial activity within this source region and/or during transport in the river. In particular, when Arctic coastal erosion releases mostly matrix-free, unprotected OM, it becomes more susceptible to strong degradation (*42*). Yedoma deposits, the major source of permafrost OM for the WESS, was formed during the Late Pleistocene. The historical thawing of Yedoma during deglacial/interglacial warming periods and its OM bioavailability are a subject of ongoing research. Its persistence in the shelf seas indicates a previously extensive decomposition of Yedoma OM, likely attributed to previous thawing. This may also be related to the fact that a larger fraction of erosional OM release is as POC as opposed to as DOC, with the former known to be more prone to degradation near the site of release and thus possibly even before export to the coastal recipient (*28*, *37*, *43*, *44*). These observations collectively suggest that the permafrost type, thawing process, and history may combine as critical determinants of decomposition status of the exported OM and, consequently, OM reactivity during cross-shelf transport.

In addition to decomposition status, the OM composition at origin profoundly influences terrestrial OM reactivity. Terrestrial OM derived from the Lena River delta exhibits high reactivity relative to other shelf seas, characterized by elevated degradation rate constant and a smaller recalcitrant fraction (Fig. 6 and Table 1). A comprehensive study on soil POM composition within the Lena River delta underscores that about 80% of the carbon stock consists of free POM and occluded POM, while the relatively stable mineral-associated OM only accounts for 20% of the total stock OC in the Lena River delta (*45*). These compositional characteristics may explain the high terrestrial OM reactivity observed in the Laptev Sea. In contrast, soil POM studies from the Lower Kolyma region and Yedoma deposits reveal that 54 ± 16% and 53 ± 20% of the total soil OM, respectively, are bound to minerals, respectively (*46*, *47*). The mineral-organic association, in addition to its stabilization of OM, tends to selectively preserve recalcitrant or microbially decomposed organic compounds (*45*, *46*), fundamentally influencing the recalcitrant fractions observed across different regimes. Consequently, the higher proportion of mineral-associated OM in the East Siberian system, compared to the Lena River delta catchment, accounts for the increased fractions of recalcitrant OC relative to those detected in the Laptev Sea.

One should recognize that beyond the inherent characteristics of OM at origin, such as its degradation status and composition, the mechanisms of supply and transport also substantially influence the type of OM delivered to the ocean and its observed reactivity in the receptor system. For example, a substantial portion of the POC transported by the Lena River is derived from the spring freshet and the erosion of Holocene bluffs in the largest Arctic delta. This material is relatively unprotected and more readily degradable, contributing to the higher reactivity observed in the Lena River transect. In contrast, although the Ob River likely exports similar fluffy material, its transport through the long Ob Bay into the open ocean appears limited, resulting in the lower reactivity observed in the Kara Sea transect.

In addition to the factors discussed above, we explored multiple other environmental factors that could potentially influence the observed patterns in carbon reactivity. We explored the potential role of bottom water temperature and O_2_ concentration. However, both parameters show minor differences across the four transects, except for a localized low-oxygen zone observed in the WESS (figs. S1 and S2). Clay mineralogy and Fe-OC associations in shelf regions may also influence the carbon reactivity. Although their broad spatial distributions cannot fully explain the patterns observed across all transects, they may help account for differences between specific shelf regions (*22*, *48*, *49*). For instance, while terrestrial OM in the Laptev Sea demonstrates enhanced reactivity, the degradation rate constant observed in the EESS is comparable or even higher than that in the Laptev Sea (Fig. 6). This may relate to differences in mineral protection. Illite, known to offer weaker organomineral binding compared to smectite, is more abundant in the Laptev and Kara seas than the WESS and EESS (*48*, *49*). The lower protective capacity of illite may help explain the elevated degradation rate in the EESS. Besides, reactive iron phases appear to play a more limited role in OM preservation in the EESS. A recent study reported that only 5.4 ± 4.0% of remobilized permafrost carbon was sequestered by reactive iron in the EESS, while the number is up to 15 ± 5.3% in the Laptev Sea (*22*). This difference may further contribute to the higher carbon reactivity observed in the EESS.

Beyond mineral protection, enhanced microbiological modulation during cross-shelf transit may also contribute to the higher carbon reactivity in the EESS. The EESS is largely influenced by Pacific water (*50*), which fosters a distinct environment in comparison to Lena River–influenced water, and this potentially affects OM dynamics. A previous study observed a greater abundance of marine labile OM [e.g., short-chain fatty acids and dicarboxylic acids from cupric oxide (CuO) oxidation] produced in the EESS in comparison to the Laptev Sea (*51*). We hypothesize that high terrestrial OM reactivity in the EESS might be linked to enhanced microbial activity and additional terrestrial OM degradation, catalyzed by more labile marine OM—a process known as priming (*52*). Karlsson *et al.* (*51*) performed incubation experiments in Laptev Sea versus East Siberian Sea and found higher relative degradation rates in the East Siberian Sea. Priming through a higher abundance of readily degradable planktonic OM in the EESS may promote enhanced reactivity of terrestrial OM exported from permafrost. However, given a lack of direct microbiological evidence, this interpretation remains speculative. Future studies incorporating direct microbiological analysis are needed to better elucidate the specific role of microbes in regulating coastal Arctic carbon cycling.

In summary, this study established an integrated framework that revealed distinct differences in reactivity of permafrost-exported OM between the four major shelf sea receptor systems, stretching across the entire northern seaboard of the Eurasian Arctic. The permafrost OM delivered to the Laptev Sea and EESS is more reactive, whereas the OM delivered to the Kara Sea and WESS is less reactive. The reactivity of exported OM is primarily determined by degradation status and composition of the expelled OM, with mineral protection and microbiological processes, e.g., priming effect, likely also playing a role. Understanding these differences in OM reactivity helps us to better comprehend the extent to which permafrost carbon in different sectors of the circum-Arctic participates in the climate-perturbed large-scale carbon cycle and improves our ability to quantify the potential impacts under changing climate.

## MATERIALS AND METHODS

### Experimental design

The study is designed to understand the geospatial patterns of terrestrial OC reactivity and their driving mechanisms across the scales of the Eurasian Arctic shelf seas. We integrated newly generated data from the Kara Sea (*n* = 25), additional gap-filling data from the WESS (*n* = 5), and published data from the Laptev Sea (*n* = 32), WESS (*n* = 9), and EESS (*n* = 9) interpreted within an integrated framework. The sample analysis includes TOC, δ^13^C, Δ^14^C, SSA, lipid biomarkers, lignin phenol biomarkers, and OC source apportionment.

### Study area and sampling

The Kara Sea receives about 50% of the total river runoff discharged to the Eurasian Arctic, with Ob River contributing 427 km^3^ year^−1^ and Yenisey River contributing 673 km^3^ year^−1^ (*35*, *53*). The supply values of particulate OC by the same rivers are 0.57 × 10^6^ and 0.25 × 10^6^ tons year^−1^, respectively (*36*, *53*). The majority of particulate material is trapped in the estuarine region, where freshwater and seawater mix and form a distinct mixing zone referred to as the “marginal filter” (*54*).

Sea ice is a key feature of the Kara Sea, which remains largely ice-covered for 8 months (October to May). Substantial amounts of sediments can be incorporated into sea ice through processes such as suspension freezing, anchor ice formation, and deposition of river sediments onto a flooded fast ice and may subsequently be exported off the shelf (*53*). A critical aspect of this process is brine formation during seawater freezing, which produces dense saline water that may flow through submarine channels and transport resuspended matter within the bottom nepheloid layer toward the outer shelf, across the shelf break, and into the Arctic basins. Active mainly from autumn to winter, this process enables sediments and OM to bypass the marginal filter (*53*).

The Kara Sea surface circulation exhibits strong seasonal variability driven by wind, river discharge, and ice formation. In spring and summer, intense river runoff induces an anticyclonic pattern, whereas in autumn and winter, circulation reverses, forming a pronounced eastward coastal current (*53*).

Surface sediments along the Ob-Kara Sea transect were obtained during the “Akademik Boris Petrov” expeditions in 1997, 1999, 2001, and 2002 and during the “International Siberian Shelf Study” expedition in 2020 (ISSS-2020). The sampling was carried out using giant box corers and multicorers, and the sediments were sectioned every centimeter and kept frozen until shore-based analyses. Surface sediments used for this study were taken from the uppermost 1 cm. Notably, during the intense runoff period, selecting a transect aligned with the major transport pathway (anticyclonic) would reduce uncertainties in the transport time estimation. However, such alignment would likely introduce a mixed signal exported from the Yenisey River, complicating the interpretation. To minimize this influence, we selected a transect extending northward from the Ob River, thereby reducing the potential Yenisey River input, although this may result in a slight overestimation of transport time.

### Carbon content and carbon isotope analyses

Freeze-dried and homogenized sediment samples were analyzed for TOC content and stable carbon isotope (δ^13^C) composition. Briefly, ~15 mg of sediment was weighed into a silver capsule and acidified with 1 M HCl for 4 hours to remove carbonates. The analytical procedures were conducted using a Flash IRMS elemental analyzer coupled to a Delta V Plus mass spectrometer.

Radiocarbon (Δ^14^C) analysis on the new samples was carried out following the method outlined by Mollenhauer *et al.* (*55*). Briefly, sediment samples containing ~1 mg of OC were weighed into silver capsules and acidified with 6 mol liter^−1^ HCl to remove inorganic carbon. Individually combusted via an Elementar Vario ISOTOPE EA (Elemental Analyzer), the oxidized carbon was graphitized using the Ionplus AGE3 system (Automated Graphitization System). The radiocarbon content was then analyzed using an Ionplus MICADAS dating system.

### SSA analyses

The approach of SSA analysis has been described by Bröder *et al.* (*56*). Approximately 1 g of freeze-dried sediment was combusted at 400°C for 12 hours to eliminate organic components, followed by overnight cooling to room temperature. The samples were subjected to a triple rinse with 50 ml of Milli-Q water to remove salts and minerals and centrifuged at 8000 rpm for 20 min, and then the overlying water was discharged before freeze-drying. Immediately preceding the analysis, the samples underwent a 2-hour degassing process under a continuous flow of N_2_ at 200°C, facilitated by a Micromeritics Flow-Prep 060 Sample Degas System. Subsequently, the samples were analyzed using a Micromeritics Gemini VII Surface Area and Porosity analyzer.

### Lipid biomarker analyses

Lipid biomarkers were analyzed following methods described in detail earlier by Bröder *et al.* (*56*). Briefly, freeze-dried and homogenized sediments were extracted through an accelerated solvent extraction (Dionex ASE 500), using a dichloromethane/methanol solvent mixture (9:1, v/v). Before extraction, five recovery standards were added, i.e., tetracosane-*d*_50_, triacontane-*d*_62_, eicosanoic acid-*d*_39_, 2-hexadecanol, and stigmasterol-*d*_5_. To eliminate sulfur, the extracts were subjected to an overnight reaction with activated copper. Subsequently, the extracts underwent separation into acid fractions and neutral fractions using aminopropyl Bond Elut. Further separation of the neutral fractions into polar fractions and nonpolar fractions was achieved through column chromatography over Al_2_O_3_. The acid and polar fractions were derivatized [*N*,*O*-bis(trimethylsilyl)trifluoroacetamide (BSTFA)+ 1% trimethylchlorosilane (TMCS), 60°C, 30 min] before instrumental analyses.

Instrumental analysis was conducted on a gas chromatograph–mass spectrometer (GC-MS) detector (Agilent GC-MS 7820A) equipped with a DB-5 column (30 m, 250-μm film, 0.3 mm), operating in splitless mode. The oven temperature program initiated at 60°C, followed by a temperature gradient of 10°C per minute until 310°C, which was maintained for 16 min. Quantification was performed by combining sample recoveries and calibration curves derived from commercially available external standards.

### CuO oxidation and lignin biomarker analyses

Methods of CuO oxidation and lignin biomarker analyses were originally presented by Goñi and Montgomery (*57*) and modified by Tesi *et al.* (*8*). Briefly, ~2 to 5 mg of OC was loaded into Teflon tubes, combined with 300 mg of CuO and 50 mg of ammonium iron(II) sulfate hexahydrate [(NH_4_)_2_Fe(SO_4_)_2_·6H_2_O], and suspended in a nitrogen-purged 2 M NaOH solution. The extraction process was performed using an Ultra WAVE milestone 215 microwave digestion system with a temperature program of 130°C for 90 min. Following the extraction, the recovery standard (ethyl-vanillin and cinnamic acid) was added, and the samples were acidified to a pH of 1. To remove residual water in the supernatant, anhydrous sodium sulfate (Na_2_SO_4_) was used, and subsequently, phenols were extracted from the supernatant using ethyl acetate (EtOAc). The resulting extracts were evaporated in a CentriVap (Christ RVC 2-25) at 60°C for 1 hour, followed by redissolution in pyridine. The extracts were derivatized (*N*,*O*-bistrifluoroacetamide + 1% trimethylsilyl chloride, 60°C, 30 min) before instrumental analyses.

The compounds were analyzed in a single ion monitoring mode on a GC-MS (Agilent GC-MS 7820A) equipped with a DB-5 capillary column (30 m, 250-μm film, 0.3 mm). The temperature program initiated at 50°C with a subsequent heating ramp of 10°C per minute until 300°C. Quantification was carried out by combining sample recoveries and calibration curves derived from commercially available external standards.

### OC source apportionment

This study used a Bayesian statistical approach to constrain the major OC sources. This followed the earlier detailed description of the method (*6*, *7*, *9*, *28*). Specifically, a three–end-member mixing model, based on δ^13^C and Δ^14^C signatures, was used to estimate the fractional contributions of the AL, ICDs, and marine OC, assuming isotopic mass balance

$$\Delta^{14}C_{\text{bulk}}=f_{\text{AL}}\times \Delta^{14}C_{\text{AL}}+f_{\text{ICD}}\times \Delta^{14}C_{\text{ICD}}+f_{\text{marine}}\times \Delta^{14}C_{\text{marine}}$$ (3)

$$\delta^{13}C_{\text{bulk}}=f_{\text{AL}}\times \delta^{13}C_{\text{AL}}+f_{\text{ICD}}\times \delta^{13}C_{\text{ICD}}+f_{\text{marine}}\times \delta^{13}C_{\text{marine}}$$ (4)

$$1=f_{\text{AL}}+f_{\text{ICD}}+f_{\text{marine}}$$ (5)

A Markov chain Monte Carlo simulation was implemented to account for uncertainties in source signatures of δ^13^C and Δ^14^C and to minimize errors from assignments of end-member values. Analytical errors of the samples were not included in the mixing model, as their influence is considered negligible compared to the end-member uncertainties. The simulation was performed in MatlabR2023a, following the script by Andersson *et al.* (*58*), encompassing 1,000,000 runs and a burn-in period of 10,000 runs per sample. Mean relative contributions and the standard deviations for the three different OC pools were thus obtained.

End-member values were defined on the basis of an extensive literature review building on the approach of previous studies in the Siberian Arctic seas (*7*, *9*, *28*). For the AL end-member, we assigned a δ^13^C value of −26.4 ± 0.8‰ and a Δ^14^C value of −198 ± 148‰ (*16*). The ICD end-member was assigned a δ^13^C value of −26.3 ± 0.7‰ and a Δ^14^C value of −962 ± 61‰ (*59*, *60*). Regarding the marine end-member, two sets of end-member values were applied to the western regime of 160°E and the eastern regime of 160°E because of a frontal zone with river-influenced waters and stronger ^13^C-depleted phytoplankton in the western East Siberian Arctic Shelf. Therefore, we assigned a δ^13^C value of −23.2 ± 3.5‰ and a Δ^14^C value of −3 ± 55‰ to the marine end-member in the western regime of 160°E, while we assigned a δ^13^C value of −21.0 ± 2.6‰ and a Δ^14^C value of −50 ± 12‰ to the marine end-member in the eastern regime of 160°E (*9*). Notably, the Ob River catchment holds the largest peatland in the world; we therefore used surface soil and subsurface peat as end-members, specifically for the Kara Sea transect source apportionment instead of AL and ICD. The surface soil was assigned a δ^13^C value of −26.6 ± 1.7‰ and a Δ^14^C value of −201 ± 229‰, whereas the subsurface peat was based on a large collection of ^14^C-dated peat cores from the circum-Arctic and was assigned a δ^13^C value of −26.6 ± 1.7‰ and a Δ^14^C value of −503 ± 159‰ (table S1) (*7*).

## Supplementary Materials

### The PDF file includes:

Figs. S1 and S2 Table S1 Legend for data S1 References
