# Supplementary material for: Geospatial patterns in terrestrial organic matter reactivity across four shelf seas spanning the Eurasian Arctic
Source: Sci Adv. 2025 Sep 10;11(37):eadt6806. doi: 10.1126/sciadv.adt6806 (PMC12422179; doi:10.1126/sciadv.adt6806)
Supplement: Supplementary file 1 — Figs. S1 and S2 Table S1 Legend for data S1 References [file sciadv.adt6806_sm.pdf]

Science Advances
AAAS

Supplementary Materials for

Geospatial patterns in terrestrial organic matter reactivity across four shelf seas spanning the Eurasian Arctic

Junjie Wu *et al.*

Corresponding author: Junjie Wu, junjie.wu@aces.su.se; Örjan Gustafsson, Orjan.Gustafsson@aces.su.se

Sci. Adv. **11**, eadt6806 (2025)
DOI: 10.1126/sciadv.adt6806

The PDF file includes:

Figs. S1 and S2
Table S1
Legend for data S1
References

Other Supplementary Material for this manuscript includes the following:

Data S1

Supplementary Figures

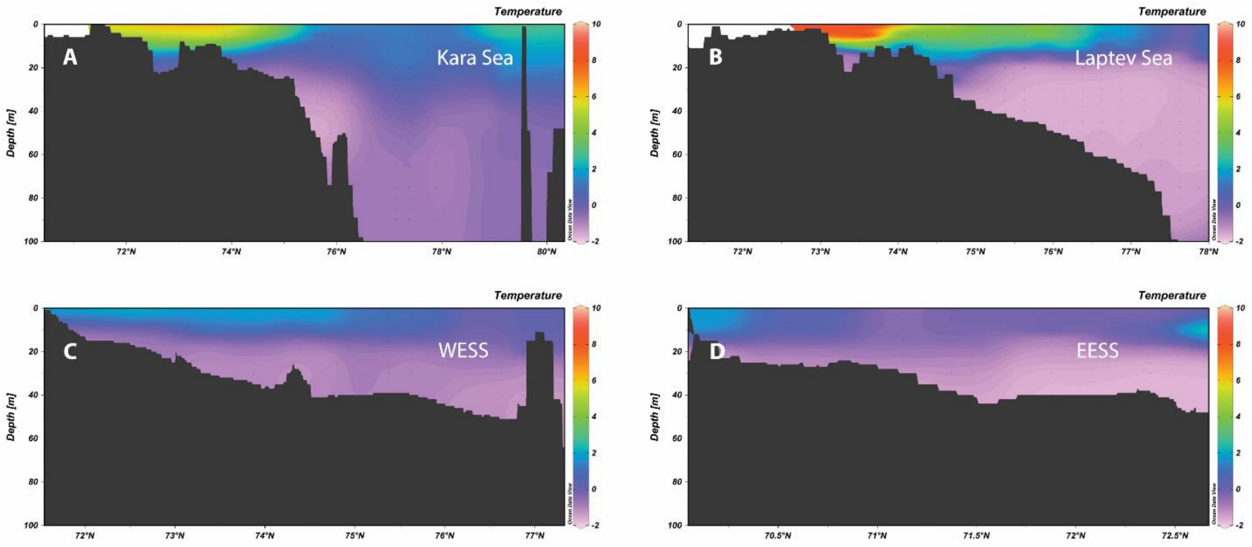

Fig. S1. Water temperature profiles for four studied transects. (**A**) temperature profile for the Kara Sea, (**B**) temperature profile for the Laptev Sea, (**C**) temperature profile for the WESS and (**D**) temperature profile for the EESS. Data are derived from World Ocean Atlas 2023 (*62*).

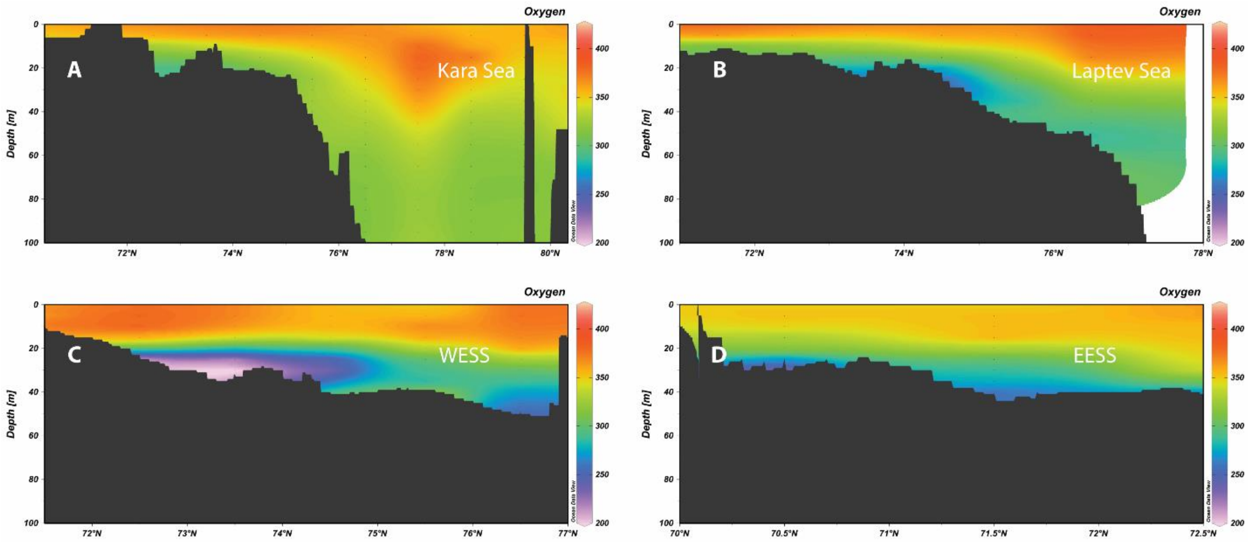

Fig. S2. Water oxygen profiles for four studied transects. (**A**) oxygen profile for the Kara Sea, (**B**) oxygen profile for the Laptev Sea, (**C**) oxygen profile for the WESS and (**D**) oxygen profile for the EESS. Data are derived from World Ocean Atlas 2023 (*63*).

Supplementary Tables

Table S1. Endmemeber values for the dual-isotope mixing model to calculate OC source fractions.

**Endmembers**	**$\Delta^{14}C$ (‰) Mean ± s.d.**	**$\delta^{13}C$ (‰) Mean ± s.d.**	**references**
Active layer OC	-198±148‰	-26.4±0.8	Wild et al., (*16*)
Ice Complex Deposits OC	-962±61	-26.3±0.7	Martens et al., (*60*); Schirrmeister et al., (*59*)
Marine OC (weast of 160°E)	-3±55	-23.2±3.5	Martens et al., (*9*)
Marine OC (east of 160°E)	-50±12	-21.0±2.6	Martens et al., (*9*)
Surface soil	-201±229	-26.6±1.7	Martens et al., (*7*)
Subsuface peat	-503±159	-26.6±1.7	Martens et al., (*7*)

Data S1. (Separate file)

The dataset comprises measurements of TOC, $\delta^{13}C$, $\Delta^{14}C$, SSA, lipid biomarkers, lignin phenol biomarkers in sediment samples, and organic carbon source apportionment results from the Kara Sea and the western East Siberian Sea, as generated by this study.

REFERENCES AND NOTES

1. G. Hugelius, J. Strauss, S. Zubrzycki, J. W. Harden, E. A. G. Schuur, C. L. Ping, L. Schirrmeister, G. Grosse, G. J. Michaelson, C. D. Koven, J. A. O'Donnell, B. Elberling, U. Mishra, P. Camill, Z. Yu, J. Palmtag, P. Kuhry, Estimated stocks of circumpolar permafrost carbon with quantified uncertainty ranges and identified data gaps. *Biogeosciences* **11**, 6573–6593 (2014).

2. P. Friedlingstein, M. O'Sullivan, M. W. Jones, R. M. Andrew, D. C. E. Bakker, J. Hauck, P. Landschützer, C. Le Quéré, I. T. Luijkx, G. P. Peters, W. Peters, J. Pongratz, C. Schwingshackl, S. Sitch, J. G. Canadell, P. Ciais, R. B. Jackson, S. R. Alin, P. Anthoni, L. Barbero, N. R. Bates, M. Becker, N. Bellouin, B. Decharme, L. Bopp, I. B. M. Brasika, P. Cadule, M. A. Chamberlain, N. Chandra, T. T. T. Chau, F. Chevallier, L. P. Chini, M. Cronin, X. Dou, K. Enyo, W. Evans, S. Falk, R. A. Feely, L. Feng, D. J. Ford, T. Gasser, J. Ghattas, T. Gkritzalis, G. Grassi, L. Gregor, N. Gruber, Ö. Gürses, I. Harris, M. Hefner, J. Heinke, R. A. Houghton, G. C. Hurtt, Y. Iida, T. Ilyina, A. R. Jacobson, A. Jain, T. Jarníková, A. Jersild, F. Jiang, Z. Jin, F. Joos, E. Kato, R. F. Keeling, D. Kennedy, K. K. Goldewijk, J. Knauer, J. I. Korsbakken, A. Körtzinger, X. Lan, N. Lefèvre, H. Li, J. Liu, Z. Liu, L. Ma, G. Marland, N. Mayot, P. C. McGuire, G. A. McKinley, G. Meyer, E. J. Morgan, D. R. Munro, S. I. Nakaoka, Y. Niwa, K. M. O'Brien, A. Olsen, A. M. Omar, T. Ono, M. Paulsen, D. Pierrot, K. Pocock, B. Poulter, C. M. Powis, G. Rehder, L. Resplandy, E. Robertson, C. Rödenbeck, T. M. Rosan, J. Schwinger, R. Séférian, T. L. Smallman, S. M. Smith, R. Sospedra-Alfonso, Q. Sun, A. J. Sutton, C. Sweeney, S. Takao, P. P. Tans, H. Tian, B. Tilbrook, H. Tsujino, F. Tubiello, G. R. van der Werf, E. van Ooijen, R. Wanninkhof, M. Watanabe, C. Wimart-Rousseau, D. Yang, X. Yang, W. Yuan, X. Yue, S. Zaehle, J. Zeng, B. Zheng, Global Carbon Budget 2023. *Earth Syst. Sci. Data* **15**, 5301–5369 (2023).

3. S. M. Natali, J. D. Watts, B. M. Rogers, S. Potter, S. M. Ludwig, A. K. Selbmann, P. F. Sullivan, B. W. Abbott, K. A. Arndt, L. Birch, M. P. Björkman, A. A. Bloom, G. Celis, T. R. Christensen, C. T. Christiansen, R. Commane, E. J. Cooper, P. Crill, C. Czimczik, S. Davydov, J. Du, J. E. Egan, B. Elberling, E. S. Euskirchen, T. Friborg, H. Genet, M. Göckede, J. P. Goodrich, P. Grogan, M. Helbig, E. E. Jafarov, J. D. Jastrow, A. A. M. Kalhori, Y. Kim, J. S. Kimball, L. Kutzbach, M. J. Lara, K. S. Larsen, B. Y. Lee, Z. Liu, M. M. Loranty, M. Lund, M. Lupascu, N. Madani, A. Malhotra, R. Matamala, J. McFarland, A. D. McGuire, A.

Michelsen, C. Minions, W. C. Oechel, D. Olefeldt, F. J. W. Parmentier, N. Pirk, B. Poulter, W. Quinton, F. Rezanezhad, D. Risk, T. Sachs, K. Schaefer, N. M. Schmidt, E. A. G. Schuur, P. R. Semenchuk, G. Shaver, O. Sonnentag, G. Starr, C. C. Treat, M. P. Waldrop, Y. Wang, J. Welker, C. Wille, X. Xu, Z. Zhang, Q. Zhuang, D. Zona, Large loss of CO_2 in winter observed across the northern permafrost region. *Nat. Clim. Change* **9**, 852–857 (2019).

4. G. Hugelius, J. Loisel, S. Chadburn, R. B. Jackson, M. Jones, G. MacDonald, M. Marushchak, D. Olefeldt, M. Packalen, M. B. Siewert, C. Treat, M. Turetsky, C. Voigt, Z. Yu, Large stocks of peatland carbon and nitrogen are vulnerable to permafrost thaw. *Proc. Natl. Acad. Sci. U.S.A.* **117**, 20438–20446 (2020).

5. M. R. Turetsky, B. W. Abbott, M. C. Jones, K. W. Anthony, D. Olefeldt, E. A. G. Schuur, G. Grosse, P. Kuhry, G. Hugelius, C. Koven, D. M. Lawrence, C. Gibson, A. B. K. Sannel, A. D. McGuire, Carbon release through abrupt permafrost thaw. *Nat. Geosci.* **13**, 138–143 (2020).

6. L. Bröder, T. Tesi, A. Andersson, I. Semiletov, Ö. Gustafsson, Bounding cross-shelf transport time and degradation in Siberian-Arctic land-ocean carbon transfer. *Nat. Commun.* **9**, 806 (2018).

7. J. Martens, B. Wild, I. Semiletov, O. V. Dudarev, Ö. Gustafsson, Circum-Arctic release of terrestrial carbon varies between regions and sources. *Nat. Commun.* **13**, 5858 (2022).

8. T. Tesi, I. Semiletov, O. Dudarev, A. Andersson, Ö. Gustafsson, Matrix association effects on hydrodynamic sorting and degradation of terrestrial organic matter during cross-shelf transport in the Laptev and East Siberian shelf seas. *J. Geophys. Res. Biogeosci.* **121**, 731–752 (2016).

9. F. Matsubara, B. Wild, J. Martens, A. Andersson, R. Wennström, L. Bröder, O. V. Dudarev, I. Semiletov, Gustafsson, Molecular-multiproxy assessment of land-derived organic matter degradation over extensive scales of the East Siberian Arctic Shelf seas. *Global Biogeochem. Cycles* **36**, e2022GB007428 (2022).

10. D. Kim, J. Kim, T. Tesi, S. Kang, A. Nogarotto, K. Park, D. Lee, Y. Keun, K. Shin, S. Nam, Changes in the burial efficiency and composition of terrestrial organic carbon along the Mackenzie Trough in the Beaufort Sea. *Estuar. Coast. Shelf Sci.* **275**, 107997 (2022).

11. B. E. van Dongen, I. Semiletov, J. W. H. Weijers, Ö. Gustafsson, Contrasting lipid biomarker composition of terrestial organic matter exported from across the Eurasian Arctic by the five great Russian Arctic rivers. *Global Biogeochem. Cycles* **22**, 10.1029/2007GB002974 (2008).

12.. Gustafsson, B. E. Van Dongen, J. E. Vonk, O. V. Dudarev, I. P. Semiletov, Widespread release of old carbon across the Siberian Arctic echoed by its large rivers. *Biogeosciences* **8**, 1737–1743 (2011).

13. K. Fahl, R. Stein, Modern organic carbon deposition in the Laptev Sea and the adjacent continental slope: Surface water productivity vs. terrigenous input. *Org. Geochem.* **26**, 379–390 (1997).

14. K. E. Grant, M. N. Repasch, K. M. Finstad, J. D. Kerr, M. Marple, C. J. Larson, T. A. B. Broek, J. Pett-Ridge, K. J. McFarlane, Diverse organic carbon dynamics captured by radiocarbon analysis of distinct compound classes in a grassland soil. *Biogeosciences* **21**, 4395–4411 (2024).

15. X. Feng, J. E. Vonk, B. E. Van Dongen, Ö. Gustafsson, I. P. Semiletov, O. V. Dudarev, Z. Wang, D. B. Montluçon, L. Wacker, T. I. Eglinton, Differential mobilization of terrestrial carbon pools in Eurasian Arctic river basins. *Proc. Natl. Acad. Sci. U.S.A.* **110**, 14168–14173 (2013).

16. B. Wild, A. Andersson, L. Bröder, J. Vonk, G. Hugelius, J. W. McClelland, W. Song, P. A. Raymond, Ö. Gustafsson, Rivers across the Siberian Arctic unearth the patterns of carbon release from thawing permafrost. *Proc. Natl. Acad. Sci. U.S.A.* **116**, 10280–10285 (2019).

17. I. Kögel-Knabner, The macromolecular organic composition of plant and microbial residues as inputs to soil organic matter. *Soil Biol. Biochem.* **34**, 139–162 (2002).

18. J. D. Hemingway, D. H. Rothman, K. E. Grant, S. Z. Rosengard, T. I. Eglinton, L. A. Derry, V. V. Galy, Mineral protection regulates long-term global preservation of natural organic carbon. *Nature* **570**, 228–231 (2019).

19. M. Kleber, I. C. Bourg, E. K. Coward, C. M. Hansel, S. C. B. Myneni, N. Nunan, Dynamic interactions at the mineral–organic matter interface. *Nat. Rev. Earth Environ.* **2**, 402–421 (2021).

20. L. M. Mayer, Surface area control of organic carbon accumulation in continental shelf sediments. *Geochim. Cosmochim. Acta* **58**, 1271–1284 (1994).

21. J. I. Hedges, R. G. Keil, Sedimentary organic matter preservation: An assessment and speculative synthesis. *Mar. Chem.* **49**, 81–115 (1995).

22. J. A. Salvadõ, T. Tesi, A. Andersson, J. Ingri, O. V. Dudarev, I. P. Semiletov, Ö. Gustafsson, Organic carbon remobilized from thawing permafrost is resequestered by reactive iron on the Eurasian Arctic Shelf. *Geophys. Res. Lett.* **42**, 8122–8130 (2015).

23. Y. Chen, L. Dong, W. Sui, M. Niu, X. Cui, K. U. Hinrichs, F. Wang, Cycling and persistence of iron-bound organic carbon in subseafloor sediments. *Nat. Commun.* **15**, 6370 (2024).

24. E. Tipping, The adsorption of aquatic humic substances by iron oxides. *Geochim. Cosmochim. Acta* **45**, 191–199 (1981).

25. M. Elmquist, I. Semiletov, L. Guo, Ö. Gustafsson, Pan-Arctic patterns in black carbon sources and fluvial discharges deduced from radiocarbon and PAH source apportionment markers in estuarine surface sediments. *Global Biogeochem. Cycles* **22**, 10.1029/2007GB002994 (2008).

26. J. A. Salvadó, L. Bröder, A. Andersson, I. P. Semiletov, Ö. Gustafsson, Release of black carbon from thawing permafrost estimated by sequestration fluxes in the East Siberian Arctic Shelf recipient. *Global Biogeochem. Cycles* **31**, 1501–1515 (2017).

27. L. Bröder, A. Andersson, T. Tesi, I. Semiletov, Ö. Gustafsson, Quantifying degradative loss of terrigenous organic carbon in surface sediments across the Laptev and East Siberian Sea. *Global Biogeochem. Cycles* **33**, 85–99 (2019).

28. J. E. Vonk, L. Sanchez-Garca, B. E. Van Dongen, V. Alling, D. Kosmach, A. Charkin, I. P. Semiletov, O. V. Dudarev, N. Shakhova, P. Roos, T. I. Eglinton, A. Andersson, A. Gustafsson,

Activation of old carbon by erosion of coastal and subsea permafrost in Arctic Siberia. *Nature* **489**, 137–140 (2012).

29. J. E. Vonk, Ö. Gustafsson, Calibrating n-alkane Sphagnum proxies in sub-Arctic Scandinavia. *Org. Geochem.* **40**, 1085–1090 (2009).

30. M. I. Behnke, S. E. Tank, J. W. McClelland, R. M. Holmes, N. Haghipour, T. I. Eglinton, P. A. Raymond, A. Suslova, A. V. Zhulidov, T. Gurtovaya, N. Zimov, S. Zimov, E. A. Mutter, E. Amos, R. G. M. Spencer, Aquatic biomass is a major source to particulate organic matter export in large Arctic rivers. *Proc. Natl. Acad. Sci. U.S.A.* **120**, e2209883120 (2023).

31. O. Ogneva, G. Mollenhauer, B. Juhls, T. Sanders, J. Palmtag, M. Fuchs, H. Grotheer, P. J. Mann, J. Strauss, Particulate organic matter in the Lena River and its delta: From the permafrost catchment to the Arctic Ocean. *Biogeosciences* **20**, 1423–1441 (2023).

32. M. B. Fernandes, M.-A. Sicre, The importance of terrestrial organic carbon inputs on Kara Sea shelves as revealed by n-alkanes, OC and d13C values. *Org. Geochem.* **31**, 363–374 (2000).

33. N. E. Blair, R. C. Aller, The fate of terrestrial organic carbon in the Marine environment. *Ann. Rev. Mar. Sci.* **4**, 401–423 (2012).

34. L. Guo, I. Semiletov, Ö. Gustafsson, J. Ingri, P. Andersson, O. Dudarev, D. White, Characterization of Siberian Arctic coastal sediments: Implications for terrestrial organic carbon export. *Global Biogeochem. Cycles* **18**, GB1036 (2004).

35. R. M. Holmes, J. W. McClelland, B. J. Peterson, S. E. Tank, E. Bulygina, T. I. Eglinton, V. V. Gordeev, T. Y. Gurtovaya, P. A. Raymond, D. J. Repeta, R. Staples, R. G. Striegl, A. V. Zhulidov, S. A. Zimov, Seasonal and annual fluxes of nutrients and organic matter from large rivers to the Arctic Ocean and surrounding seas. *Estuaries Coasts* **35**, 369–382 (2012).

36. J. W. McClelland, R. M. Holmes, B. J. Peterson, P. A. Raymond, R. G. Striegl, A. V. Zhulidov, S. A. Zimov, N. Zimov, S. E. Tank, R. G. M. Spencer, R. Staples, T. Y. Gurtovaya,

C. G. Griffin, Particulate organic carbon and nitrogen export from major Arctic rivers. *Global Biogeochem. Cycles* **30**, 629–643 (2016).

37. B. E. van Dongen, Z. Zencak, Ö. Gustafsson, Differential transport and degradation of bulk organic carbon and specific terrestrial biomarkers in the surface waters of a sub-arctic brackish bay mixing zone. *Mar. Chem.* **112**, 203–214 (2008).

38. L. Sánchez-García, V. Alling, S. Pugach, J. Vonk, B. Van Dongen, C. Humborg, O. Dudarev, I. Semiletov, Ö. Gustafsson, Inventories and behavior of particulate organic carbon in the Laptev and East Siberian seas. *Global Biogeochem. Cycles* **25**, 10.1029/2010GB003862 (2011).

39. D. Jong, L. Bröder, T. Tesi, K. H. Keskitalo, N. Zimov, A. Davydova, P. Pika, N. Haghipour, T. I. Eglinton, J. E. Vonk, Contrasts in dissolved, particulate, and sedimentary organic carbon from the Kolyma River to the East Siberian Shelf. *Biogeosciences* **20**, 271–294 (2023).

40. K. Attermeyer, N. Catalán, K. Einarsdottir, A. Freixa, M. Groeneveld, J. A. Hawkes, J. Bergquist, L. J. Tranvik, Organic carbon processing during transport through boreal inland waters: Particles as important sites. *J. Geophys. Res. Biogeosci.* **123**, 2412–2428 (2018).

41. C. C. Treat, T. Kleinen, N. Broothaerts, A. S. Dalton, R. Dommaine, T. A. Douglas, J. Z. Drexler, S. A. Finkelstein, G. Grosse, G. Hope, J. Hutchings, M. C. Jones, P. Kuhry, T. Lacourse, O. Lähteenoja, J. Loisel, B. Notebaert, R. J. Payne, D. M. Peteet, A. B. K. Sannel, J. M. Stelling, J. Strauss, G. T. Swindles, J. Talbot, C. Tarnocai, G. Verstraeten, C. J. Williams, Z. Xia, Z. Yu, M. Väliranta, M. Hättestrand, H. Alexanderson, V. Brovkin, Widespread global peatland establishment and persistence over the last 130,000 y. *Proc. Natl. Acad. Sci. U.S.A.* **116**, 4822–4827 (2019).

42. F. C. J. van Crimpen, L. Madaj, D. Whalen, T. Tesi, J. M. van Genuchten, L. Bröder, T. I. Eglinton, N. Haghipour, J. E. Vonk, Traveling light: Arctic coastal erosion releases mostly matrix free, unprotected organic carbon. *Geophys. Res. Lett.* **51**, e2024GL108622 (2024).

43. L. Sánchez-García, J. E. Vonk, A. N. Charkin, D. Kosmach, O. V. Dudarev, I. P. Semiletov, O. Gustafsson, Characterisation of three regimes of collapsing arctic ice complex deposits on

the SE Laptev Sea coast using biomarkers and dual carbon isotopes. *Permafr. Periglac. Process* **25**, 172–183 (2014).

44. O. Gustafsson, A. Widerlund, P. S. Andersson, J. Ingri, A. Ledin, Colloid dynamics and transport of major elements through a boreal river-brackish bay mixing zone. *Mar. Chem.* **71**, 1–21 (2000).

45. I. Prater, S. Zubrzycki, F. Buegger, L. C. Zoor-Füllgraff, G. Angst, M. Dannenmann, C. W. Mueller, From fibrous plant residues to mineral-associated organic carbon - The fate of organic matter in Arctic permafrost soils. *Biogeosciences* **17**, 3367–3383 (2020).

46. N. Gentsch, R. Mikutta, O. Shibistova, B. Wild, J. Schnecker, A. Richter, T. Urich, A. Gittel, H. Šantrůčková, J. Bárta, N. Lashchinskiy, C. W. Mueller, R. Fuß, G. Guggenberger, Properties and bioavailability of particulate and mineral-associated organic matter in Arctic permafrost soils, Lower Kolyma Region, Russia. *Eur. J. Soil Sci.* **66**, 722–734 (2015).

47. J. Martens, C. Rosinger, C. W. Mueller, P. Joshi, Stabilization of mineral-associated organic carbon in Pleistocene permafrost. *Nat. Commun.* **14**, 2120–2128 (2023).

48. W. B. Myers, D. A. Darby, A compilation of the silt and clay mineralogy from coastal and shelf regions of the Arctic Ocean. *Mar. Geol.* **454**, 106948 (2022).

49. R. Stein, *Arctic Ocean Sediments: Processes, Proxies, and Paleoenvironment* (Elsevier, 2008).

50. I. Semiletov, O. Dudarev, V. Luchin, A. Charkin, K. H. Shin, N. Tanaka, The East Siberian Sea as a transition zone between Pacific-derived waters and Arctic shelf waters. *Geophys. Res. Lett.* **32**, L10614 (2005).

51. E. S. Karlsson, V. Brüchert, T. Tesi, A. Charkin, O. Dudarev, I. Semiletov, O. Gustafsson, Contrasting regimes for organic matter degradation in the East Siberian Sea and the Laptev Sea assessed through microbial incubations and molecular markers. *Mar. Chem.* **170**, 11–22 (2015).

52. P. van Nugteren, L. Moodley, G. J. Brummer, C. H. R. Heip, P. M. J. Herman, J. J. Middelburg, Seafloor ecosystem functioning: The importance of organic matter priming. *Mar. Biol.* **156**, 2277–2287 (2009).

53. R. Stein, R. W. Macdonald, *The Organic Carbon in the Arctic Ocean* (Springer Berlin, Heidelberg, 2004).

54. A. P. Lisitzin, "The continental-ocean boundary as a marginal filter in the world oceans" in *Biogeochemical Cycling and Sediment Ecology* (Springer, Dordrecht, 1999), pp. 69–103.

55. G. Mollenhauer, H. Grotheer, T. Gentz, E. Bonk, J. Hefter, Standard operation procedures and performance of the MICADAS radiocarbon laboratory at Alfred Wegener Institute (AWI), Germany. *Nucl. Instrum. Methods Phys. Res. B* **496**, 45–51 (2021).

56. L. Bröder, T. Tesi, A. Andersson, T. I. Eglinton, I. P. Semiletov, O. V. Dudarev, P. Roos, Ö. Gustafsson, Historical records of organic matter supply and degradation status in the East Siberian Sea. *Org. Geochem.* **91**, 16–30 (2016).

57. M. A. Goñi, S. Montgomery, Alkaline CuO oxidation with a microwave digestion system: Lignin analyses of geochemical samples. *Anal. Chem.* **72**, 3116–3121 (2000).

58. A. Andersson, J. Deng, K. Du, M. Zheng, C. Yan, M. Sköld, Ö. Gustafsson, Regionally-varying combustion sources of the January 2013 severe haze events over Eastern China. *Environ. Sci. Technol.* **49**, 2038–2043 (2015).

59. L. Schirrmeister, V. Kunitsky, G. Grosse, S. Wetterich, H. Meyer, G. Schwamborn, O. Babiy, A. Derevyagin, C. Siegert, Sedimentary characteristics and origin of the Late Pleistocene Ice Complex on north-east Siberian Arctic coastal lowlands and islands - A review. *Quat. Int.* **241**, 3–25 (2011).

60. J. Martens, B. Wild, F. Muschitiello, M. O. Regan, M. Jakobsson, I. Semiletov, O. V. Dudarev, Ö. Gustafsson, Remobilization of dormant carbon from Siberian-Arctic permafrost during three past warming events. *Sci. Adv.* **6**, eabb6546 (2020).

61. M. Jakobsson, L. A. Mayer, C. Bringensparr, C. F. Castro, R. Mohammad, P. Johnson, T. Ketter, D. Accettella, D. Amblas, L. An, J. E. Arndt, M. Canals, J. L. Casamor, N. Chauché, B. Coakley, S. Danielson, M. Demarte, M. L. Dickson, B. Dorschel, J. A. Dowdeswell, S. Dreutter, A. C. Fremand, D. Gallant, J. K. Hall, L. Hehemann, H. Hodnesdal, J. Hong, R. Ivaldi, E. Kane, I. Klaucke, D. W. Krawczyk, Y. Kristoffersen, B. R. Kuipers, R. Millan, G. Masetti, M. Morlighem, R. Noormets, M. M. Prescott, M. Rebesco, E. Rignot, I. Semiletov, A. J. Tate, P. Travaglini, I. Velicogna, P. Weatherall, W. Weinrebe, J. K. Willis, M. Wood, Y. Zarayskaya, T. Zhang, M. Zimmermann, K. B. Zinglersen, The International Bathymetric Chart of the Arctic Ocean Version 4.0. *Sci. Data* **7**, 176 (2020).

62. R. A. Locarnini, A. V. Mishonov, O. K. Baranova, J. R. Reagan, T. P. Boyer, D. Seidov, Z. Wang, H. E. Garcia, C. Bouchard, S. L. Cross, C. R. Paver, D. Dukhovskoy, World Ocean Atlas 2023, Volume 1: Temperature, A. Mishonov, Ed. (NOAA Atlas NESDIS 89, 2024), p. 52.

63. H. E. Garcia, Z. Wang, C. Bouchard, S. L. Cross, C. R. Paver, J. R. Reagan, T. P. Boyer, R. A. Locarnini, A. V. Mishonov, O. Baranova, D. Seidov, D. Dukhovskoy, World Ocean Atlas 2023, Volume 3: Dissolved Oxygen, Apparent Oxygen Utilization, and Oxygen Saturation, A. Mishonov, Ed. (NOAA Atlas NESDIS 91, 2024).
